# Supplementary material for: Patterns of Intron Gain and Loss in Fungi
Source: PLoS Biol. 2004 Nov 30;2(12):e422. doi: 10.1371/journal.pbio.0020422 (PMC532390; doi:10.1371/journal.pbio.0020422)
Supplement: Table S1 — Also available at http://genes.mit.edu/NielsenEtAl/. (4.3 MB ZIP). [file pbio.0020422.st001.zip › NielsenEtAl/html/532.html]

AN3163.1.NCU03388.1.MG02410.1.FG10909.1


```
 CLUSTAL W (1.82) Multiple Sequence Alignments - Introns Inserted


Sequence 1: NCU03388.1	371 aa
Sequence 2: MG02410.1	360 aa
Sequence 3: FG10909.1	369 aa
Sequence 4: AN3163.1	300 aa
Alignment Length: 380 aa
Number Identitical Residues: 176 aa
Alignment Score (without introns) 8007


MG02410.1 	MSTNT---TAQ--LNGQEGSVGSSR-----GKAPDGGETQIGFRPQTNMSVQPPRQEDLQ
NCU03388.1	MSSIS---SDQPALKGK-GPVPNYD-----HHHESTANGAGGFRPQAPMAVQPPKAEDLQ
FG10909.1 	MSSTEGMINGGSSTANSKAPAPAYDPEHTTEHTHASTEGNGGFISQNKMTVVPPKKEDLQ
AN3163.1  	MSAES---------------STAIN-----TAANTGVNGKATQQPHGLVEVQPPRLSDLQ
          	**:                                  :      .:  : * **: .***

MG02410.1 	KSYASIVGDEANPKGWYGTMV1NAFGSVIGTLGAFPCCICCPNPYKNVQQGNVGLVTKFG
NCU03388.1	KSYASIITNDADPKDWYGYMI~NGLGAVIGTIGAIPCCIMCPNPYKTVEQGNVGLVTKFG
FG10909.1 	RKYATVVEQEAGDNDWYGGMM1NVLGGIVGTMGAIPCCIICPNPYKEVHQGNVGLVTKFG
AN3163.1  	PRYASTIEHDDSNPEAHG---~--------------------------------------
          	  **: : .: .    :*                                          

MG02410.1 	RFYKAVD~PGLVKINPLSERLVQVDVKIQIVEVPKQVCMTKDNVTLHLTSVIYYHIVSPH
NCU03388.1	KFYKAVD~PGLVRVNPLAEKLIQVDVKIQIVEVPQQVCMTKDNVTVQLTSVIYYHIVSPH
FG10909.1 	KFYKAVD~PGLVKINPLSERLLQIDVKIQTTEVPEQICMTKDNVTLRLTSVIYYHIVSPH
AN3163.1  	-WYASFN1PGLVKVNPLSERLITIDVKIQIVEVPRQICMTKDNVTLNLTSVIYYQVVSPH
          	 :* :.: ****::***:*:*: :***** .***.*:********:.*******::****

MG02410.1 	KAAFGIANVRQALVERTQTTLRHVVGARVLQDVIERREEVAQSIGEIIEDVAAGWGVQVE
NCU03388.1	KAAFGITNVKQALIERTQTTLRHVIGARVLQDVIERREEIAQSIGEIIEDVAAEWGVAVE
FG10909.1 	KAAFGINNVKQALMERTQTTLRHVVGARVLQDVIERREEIAQSIGEIIEDVAAGWGVQVE
AN3163.1  	KAAFGISNIKQALVERTQTTLRHVIGARVLQDVIERREEIAQSTSEIIEEVASGWGVNVE
          	****** *::***:**********:**************:*** .****:**: *** **

MG02410.1 	SMLIKDIIFSQELQESLSMAAQSKRIGESKIIAAKAE~VESAKLMRR~AADVLSSGPAMQ
NCU03388.1	SMLIKDIIFSHELQDSLSMAAQSKRIGESKIIAAKAE~VEAAKLMRQ~AADILSSAPAMQ
FG10909.1 	SMLIKDIVFSQELQESLSMAAQSKRIGESKIIAAKAE0VESAKLMRQ~AADILSSAPAMQ
AN3163.1  	SMLIKDIIFSDDLQDSLSMAAQSKRIGESKVIAARAE~VESAKLMRQ0AADILSSAPAMQ
          	*******:**.:**:***************:***:** **:*****: ***:***.****

MG02410.1 	IRYLEAMQAMAKSANSKVIFLPATNQTIP-DINPNPNLNP----NPDYNGEG-GS----S
NCU03388.1	IRYLEAMQAMAKSANSKVIFLPATNQTMPSQAQFNASLDAPGSFNPGEQGEGSGSKNKTS
FG10909.1 	IRYLEAMQAMAKSANSKVIFLPGASQSMG--GALNAALTN-------ETGESSGN--NFG
AN3163.1  	IRYLEAMQAMAKTANSKVIFLPAPNQTVQ--QQLAAAENA---------GEGPSR-----
          	************:*********...*::       .             **...      

MG02410.1 	PFHFGDTQDPGYQQALQARMVENI
NCU03388.1	TFHDFGGSDGGFQQALNAHIVENL
FG10909.1 	GQQDFGGQDPGFQQAMNARVVENI
AN3163.1  	-YAPPQGGD-GFQHAINARVVEDI
          	        * *:*:*::*::**::
```
